# The clinical value of IL-3, IL-4, IL-12p70, IL17A, IFN-γ, MIP-1β, NLR, P-selectin, and TNF-α in differentiating bloodstream infections caused by gram-negative, gram-positive bacteria and fungi in hospitalized patients: An Observational Study

**DOI:** 10.1097/MD.0000000000017315

**Published:** 2019-09-20

**Authors:** Xinjun Li, Xiaozhou Yuan, Chengbin Wang

**Affiliations:** Department of Clinical Laboratory Medicine, Chinese PLA General Hospital & Medical School of Chinese PLA, Beijing, China.

**Keywords:** biological markers, bloodstream infection, fungi, gram-negative bacteria, gram-positive bacteria

## Abstract

Early differential diagnosis of bloodstream infections (BSIs) caused by different sources and species of bacteria in hospitalized patients is crucial for the timely targeted interventions including appropriate use of antibiotics. The aim of this study was to identify 9 biomarkers for the early differentiation of gram-negative-bloodstream infection (GN-BSI), gram-positive (GP)-BSI, and fungal-BSI.

A prospective study was conducted for a total of 390 inpatients who underwent blood culture in the Chinese PLA General Hospital from September 2015 to March 2018. Patients with positive culture of a single pathogen were divided into GN-BSI, GP-BSI, and Fungal-BSI groups, and a culture-negative disease control group was also established. The serum levels of macrophage inflammatory protein 1β (MIP-1β), tumor necrosis factor α (TNF-α), interleukin (IL)-3, interferon (IFN)-γ, IL-17A, IL-4, IL-12p70, and P-selectin were detected and the NLR was calculated from routine blood test. Receiver-operating characteristic analysis was used to determine the efficacy of various indicators in the differential diagnosis of BSIs. Prediction and validation experiments on clinical patient samples (263 cases) were also performed.

The level of IL-3 in the GP-BSI group was significantly higher than those in the other 3 groups. The level of IFN-γ in the fungal-BSI group was significantly higher than those in the other 3 groups. NLR, MIP-1β, TNF-α, IL-17A, and IL3 exhibited some efficacy when distinguishing between GN-BSI and GP-BSI and NLR had the largest area under curve (AUC) (0.728), followed by MIP-1β with an AUC of 0.679. IFN-γ and IL-3 exhibited some value in differential diagnosis between GN-BSI and Fungal-BSI. IL-3, MIP-1β, TNF-α, IFN-γ, NLR, IL-17A, and IL-4 exhibited some value in distinguishing fungal-BSI and GP-BSI, with IL-3 had the largest AUC (0.722), followed by MIP-1β with an AUC of 0.703.

NLR and MIP-1β may be valuable in differentiating GN-BSI from GP-BSI in hospitalized patients. IFN-γ and IL-3 may be helpful in differential diagnosis GN-BSI and fungal-BSI. IL-3 and MIP-1β exhibited some diagnostic efficacy in distinguishing fungal-BSI and GP-BSI. Additionally, IL-3 with high serum level may be a marker for GP-BSI and IFN-γ with high serum level may be a valuable marker for the prediction of Fungal-BSI. The utility of these biomarkers to predict BSIs owing to different pathogens in hospitalized patients needs to be assessed in further studies.

## 1 Background

A bloodstream infection (BSI) refers to a condition in which pathogens are persistently present and rapidly multiply after invasion of the circulatory system, causing systemic chills, fever, hepatosplenomegaly, and rashes. Without timely control, a BSI can have a poor prognosis, further deteriorate into sepsis or septic shock, and even cause death in patients. BSI is a common infectious disease in hospitals^[1,2]^ and the infectious types mainly include gram-negative BSI (GN-BSI), gram-positive BSI (GP-BSI) and fungal-BSI.^[3,4]^

Currently, blood culture is still the criterion standard for the diagnosis of bacterial BSI.^[5]^ However, blood culture requires a long time, and the sensitivity and positive rate are low, which does not allow early diagnosis of and clinical guidance for BSIs. In addition, although matrix-assisted laser desorption/ionization time of flight mass spectrometry (MALDI-TOF-MS) has been initially used for the auxiliary diagnosis of pathogens in BSIs^[6,7]^ and the reporting time of the results is earlier than that of blood culture, but MALDI-TOF-MS is based on blood culture method and so has certain limitations in the early diagnosis of pathogens in BSIs. Other diagnostic methods for BSI include real-time quantitative PCR and peptide nucleic acid fluorescence in situ hybridization.^[8–10]^ But the extraction process of bacterial DNA and the whole experimental operation are complicated and time-consuming. Therefore, early, rapid, accurate, and reliable differential diagnosis of different types of BSIs needs to be explored, and then, targeted treatment could be achieved in most BSI patients.

When the pathogen enters the circulatory system, it interacts with the body, thereby promoting immune cells to produce a series of serum markers, including cytokines/chemokines and acute phase reaction proteins. The value of these markers for early auxiliary diagnosis of BSI and differentiation of BSIs among different types of pathogens have been investigated.^[11,12]^ Studies have shown that the concentrations of cytokines/chemokines may increase earlier than those of existing infectious inflammatory markers such as C-reactive protein and procalcitonin, and cytokines/chemokines may have a certain value in the early diagnosis or differentiation of GN-BSI, GP-BSI, and fungal-BSI.^[13]^ Therefore, in this study, we focused on 7 inflammatory-related cytokines/chemokines, including interleukin(IL)-3, IL-4, IL-12p70, IL-17A, interferon-γ (IFN-γ), macrophage inflammatory protein 1β (MIP-1β), and tumor necrosis factor α (TNF-α), and 2 other inflammatory markers including neutrophil lymphocyte ratio (NLR) and P-selectin, to evaluate the value of these markers in distinguishing among GN-BSI, GP-BSI, and fungal-BSI. This study aimed to identify serum components that can be used as auxiliary markers for the early diagnosis of BSIs and for differentiation of GN-BSI, GP-BSI, and fungal-BSI, thus providing the basis for early differential diagnosis of various types of BSIs.

## 2 Material and methods

### 2.1 Study subjects

The study included hospitalized patients in Chinese PLA General Hospital who underwent blood culture between September 2015 and March 2018. Informed consent was obtained from all subjects enrolled. This study was reviewed and approved by the Ethics Committee of Chinese PLA General Hospital (S2018-207-02) and was performed in accordance with the Helsinki Declaration.

### 2.2 Collection of early serum samples from BSI patients

Blood samples were collected from all the enrolled patients. Positive BSI specimens included serum specimens with positive results from 2 consecutive clinical blood cultures in which the 2 blood culture-positive pathogens were the same. The pathogens were identified by VITEK MS system (BioMérieux, France) and VITEK 2 Compact (BioMérieux, France). The diagnostic criteria of BSIs were based on the 1992 American College of Chest Physicians/Society of Critical Care Medicine (ACCP/SCCM) consensus conference standard and the International Guidelines for Management of Severe Sepsis and Septic Shock: 2012 and 2016.^[14]^ All patients underwent blood collection during the early stage of onset (within 48 hours of admission) and before the use of antibiotics. Blood specimens from patients who underwent blood culture were centrifuged at 3000 rpm for 5 minutes and aliquoted in sterile EP tubes. The samples were stored at −80°C for testing.

### 2.3 Study groups

Patients with positive blood culture results were divided into GN-BSI, GP-BSI, and fungal-BSI groups according to the type of pathogen present, and a blood culture-negative disease control group was also established. The inclusion criteria for each group were strictly defined. Except for the BSI, patients with primary infection had no other focus of infection, including venous catheter-related infections; patients with secondary infection had foci of infection other than BSI, but the isolated pathogens were consistent with blood culture results. Pregnant patients, patients with liver dysfunction, organ transplantation patients, patients who received immunosuppressive agents, and patients with autoimmune diseases were excluded.

### 2.4 Detection of serum IL-3, IL-4, IL-12p70, IL-17A, IFN-γ, MIP-1β, P-selectin, and TNF-α levels

Before testing, the collected serum samples were first thawed, mixed uniformly, and centrifuged, and the supernatant was collected for testing. The concentrations of the IL-3, IL-4, IL-12p70, IL-17A, IFN-γ, MIP-1β, P-selectin, and TNF-α in the serum were detected using a Milliplex MAP Magnetic Bead Panel based on the Luminex xMAP System (Milliplex, Germany). The assays were conducted in strict accordance with the operating instructions of the instrument, and the data were analyzed using Milliplex analyte v5.1.0.0 software.

### 2.5 Obtainment of the NLR

NLR was calculated based on blood routine indexes. Peripheral venous blood samples from fasting patients in each group were obtained within 48 hours of admission and before the use of antibiotics. Routine blood tests were measured using Sysmex XE-5000 5-classification blood-counter system (Sysmex, Japan).

### 2.6 Statistical analysis

Data were processed using SPSS 22.0 (SPSS Inc, Chicago, IL). For normally distributed data, the mean and standard deviation were used to represent concentrated and discrete trends, a *t* test was used to compare data between 2 groups, and analysis of variance was used to compare data among multiple groups. For non-normally distributed data, the median and quartiles were used to represent concentrated and discrete trends, and a nonparametric test was used to compare data between ≥2 groups. Receiver-operating characteristic (ROC) curve analysis was used to determine the significance of various indicators in the differential diagnosis of BSIs because of various types of pathogens. A value of *P* < .05 was regarded as statistically significant.

## 3 Results

### 3.1 Pathogen groups and the distribution of patients among hospital departments

During the study period, serum was collected from a total of 5233 inpatients who underwent blood culture. Based on the final blood culture results, 94 cases of mixed infection and 78 cases of culture contamination were excluded. A total of 673 cases (382 males vs 291 females, age 53.25 ± 21.50 years) with positive culture of a single pathogen were included in the study. According to the grouping principle, we selected 319 patients (176 male vs 143 female, age 55.25 ± 21.75 years) with a single-pathogen BSI for inclusion in the GN-BSI group (147 cases) (78 males vs 69 females, age 53.75 ± 20.75 years), GP-BSI group (123 cases) (67 males vs 56 females, age 56.25 ± 19.25 years), and fungal-BSI group (49 cases) (31 male vs 18 female, age 54.25 ± 20.50 years). A total of 71 cases (38 males vs 33 females, 55.50 ± 20.25 years) with negative culture results were randomly selected for the culture-negative control group. No significant differences in age, sex, or other basic data were observed among the groups (*P =* .335).

Patients with blood culture-positive pathogens were mainly treated in the Department of Respiratory Medicine (91/319, 28.5%), Department of Hepatobiliary Surgery (85/319, 26.6%), Department of Gastroenterology (30/319, 9.4%), Department of Cardiology (27/319, 8.5%), Department of Urology (19/319, 6.0%), and Emergency Department (18/319, 5.6%). GN bacteria were mainly isolated from Department of Hepatobiliary Surgery (55/147, 37.4%), Department of Respiratory Medicine (42/147, 28.6%), and Department of Gastroenterology(11/147, 7.5%). GP bacteria were mainly isolated from Department of Respiratory Medicine (44/123, 35.8%), Department of Hepatobiliary Surgery (17/123, 13.8%), Department of Cardiology (13/123, 10.6%), and Department of Neurosurgery (13/123, 10.6%). Fungi were mainly isolated from Department of Hepatobiliary Surgery (13/49, 26.5%), Department of Gastroenterology (11/49, 22.4%), and Department of Cardiology (10/49, 20.4%) (Table 1).

**Table 1:**
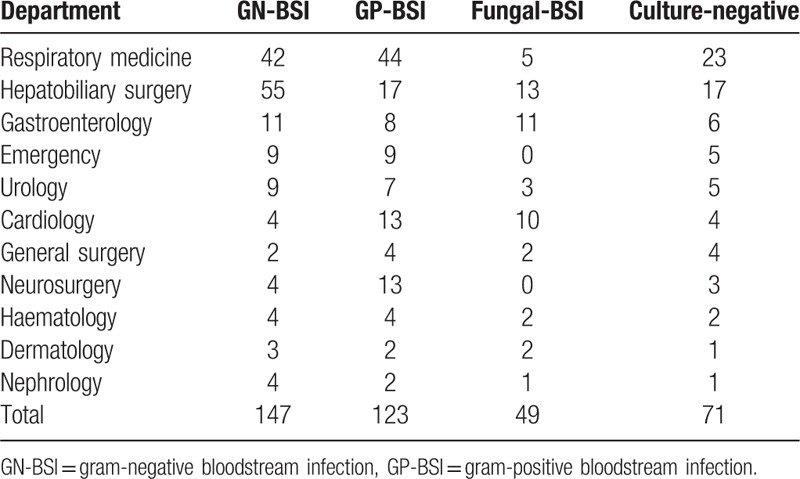
Pathogen distribution of enrolled patients among hospital departments.

### 3.2 Species distribution of pathogens isolated from patients with BSIs

Among the pathogens detected by blood culture, *Escherichia coli* had the highest positivity rate of 60 of 319 (18.8%), followed by *Klebsiella pneumoniae* (36/319, 11.3%), *Staphylococcus hominis* (31/319, 9.7%), *Staphylococcus aureus* (27/319, 8.5%), and *Acinetobacter baumannii* (26/319, 8.2%). Five fungi were isolated from the fungal-BSI group, including *Candida albicans, Candida glabrata, Candida tropicalis*, *Candida parapsilosis*, and *Candida lusitaniae*. The details of the distribution of pathogen species are shown in Table 2.

**Table 2:**
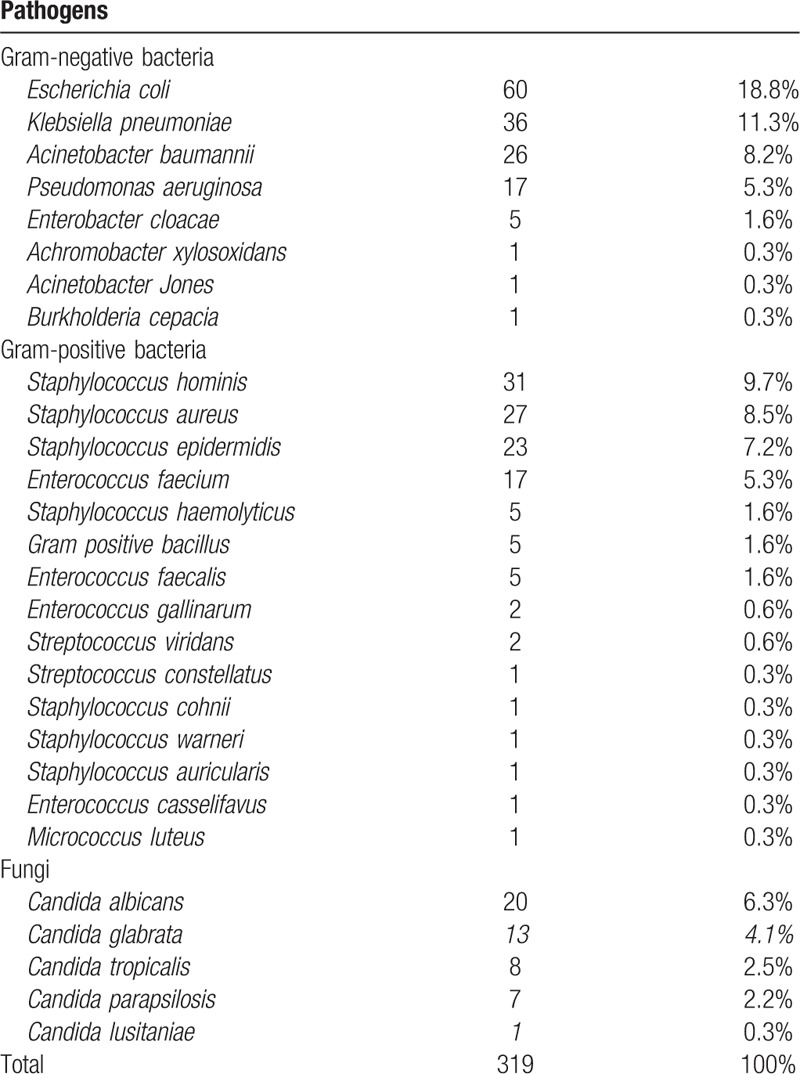
Species distribution of pathogens isolated from patients with bloodstream infections.

### 3.3 Levels of the nine biomarkers

**Figure 1:**
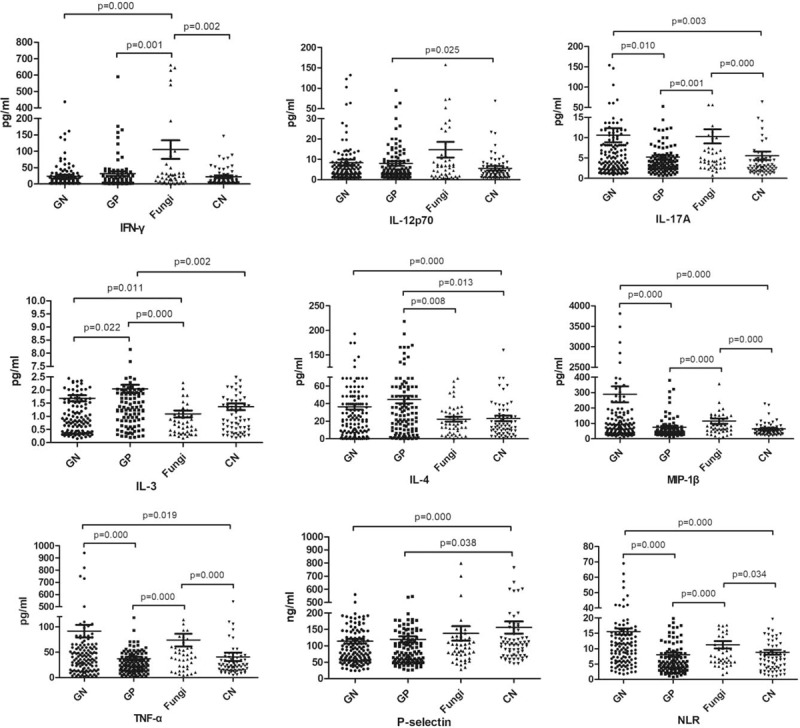
Differences of 9 biomarkers in serum samples of enrolled patients. CN = culture negative, GN = gram-negative, GP = gram-positive.

The levels of the 9 biomarkers in patients in the GN-BSI, GP-BSI, and fungal-BSI groups are shown in Table 3. The level of IL-3 in the GP-BSI group was significantly higher than those in the other 3 groups (*P* = .022, *P* < .001, and *P* = .002). The level of IFN-γ in the fungal-BSI group was significantly higher than those in the other 3 groups (*P* < .001, *P* = .001, and *P* = .002). The levels of NLR, MIP-1β, TNF-α, IL-17A in the GN-BSI group were significantly higher than those in the GP-BSI group (*P* < .001, *P* < .001, *P* < .001, and *P* = .010). The IL-3 level was significantly higher in the GN-BSI group than that in the Fungal-BSI group (*P* = .011) and the level of IFN-γ in the fungal-BSI group was significantly higher than that in the GN-BSI group (*P* < .001). The levels of MIP-1β, TNF-α, IFN-γ, NLR, IL-17A in the fungal-BSI group were significantly higher than those in the GP-BSI group (*P* < .001, *P* < .001, *P* = .001, *P* < .001, and *P* = .001) and the levels of IL-3 and IL-4 were significantly higher in GP-BSI group than in fungal-BSI group (*P* < .001 and *P* = .008). The P-selectin and IL-12p70 levels were not significantly different among GN-BSI, GP-BSI, and fungal-BSI groups (*P* > .05) (Table 4, Fig. 1). Therefore, we focused on 7 factors (IL-3, IL-4, MIP-1β, TNF-α, IFN-γ, NLR, and IL-17A) for the subsequent study of the differential diagnosis of BSIs owing to different pathogen types.

**Table 3:**
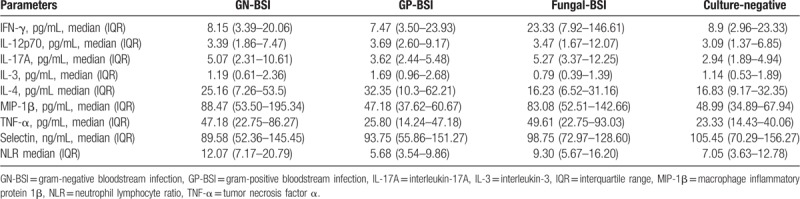
Levels of 9 biomarkers in the serum of enrolled patients.

**Table 4:**
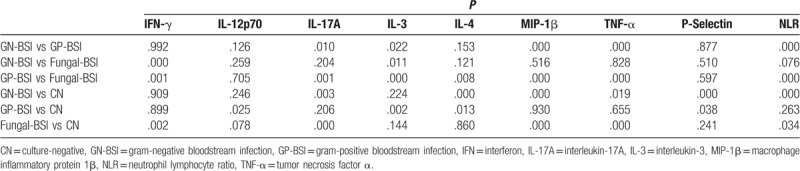
The significance of the difference (*P* value) of the 9 biomarkers’ levels between any 2 groups.

### 3.4 Values of indicators in the differential diagnosis of BSIs owing to different pathogens according to ROC analysis

**Figure 2:**
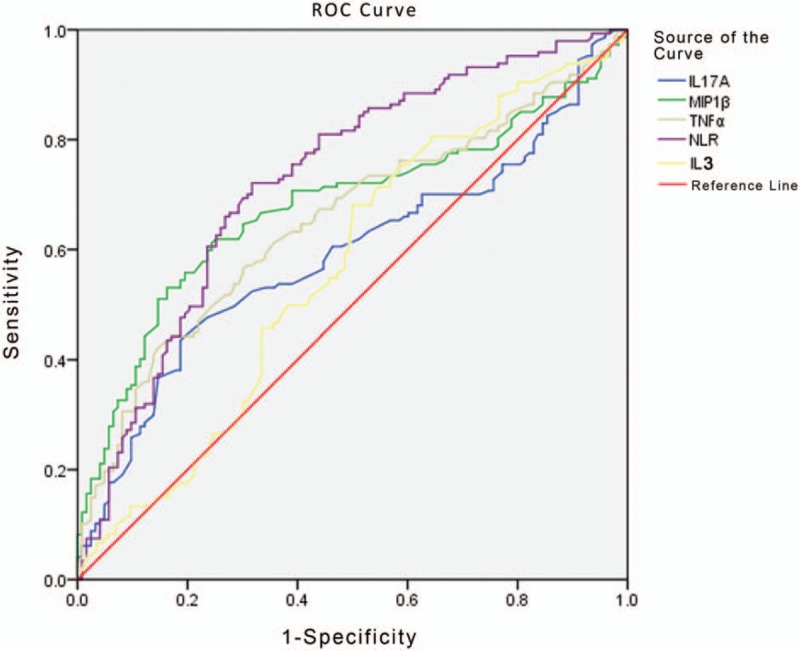
ROC curve analysis showing the differential diagnosis performance of NLR, MIP-1β, TNF-α, IL-17A, and IL-3 in discriminating GN-BSI from GP-BSI. The AUCs of NLR, MIP-1β, TNF-α, IL-17A, and IL-3 were 0.728, 0.679, 0.653, 0.591, and 0.581, respectively. GN-BSI = gram-negative bloodstream infection, GP-BSI = gram-positive bloodstream infection, IL-17A = interleukin-17A, IL-3 = interleukin-3, MIP-1β = macrophage inflammatory protein 1β, NLR = neutrophil lymphocyte ratio, ROC = receiver-operating characteristic, TNF-α = tumor necrosis factor α.

Five markers (IL-3, NLR, MIP-1β, TNF-α, and IL-17A) were selected for examination of their value in the differential diagnosis between GN-BSI and GP-BSI (Fig. 2). The analysis showed that when the cutoff value of NLR was 8.25, the AUC was 0.728, and the sensitivity and specificity were 72.1% and 68.3%, respectively. The AUC for NLR was greater than those for MIP-1β (AUC 0.679, cut-off = 85.73 pg/mL), TNF-α (AUC 0.653, cut-off = 54.18 pg/mL), IL-17A (AUC 0.591, cut-off = 6.38 pg/mL), and IL-3 (AUC 0.581, cut-off = 1.2 pg/mL). The area under curve (AUC), sensitivity (Se), specificity (Sp), negative predictive value, positive predictive value, and Youden Index when distinguishing GN-BSI and GP-BSI using various markers are shown in Table 5.

**Table 5:**
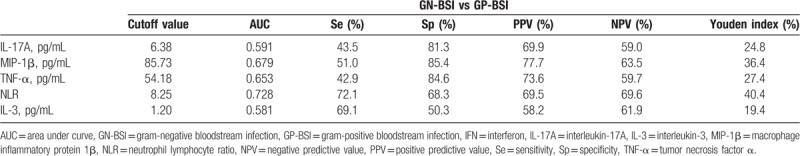
Values of biomarkers in discriminating GN-BSI from GP-BSI.

**Figure 3:**
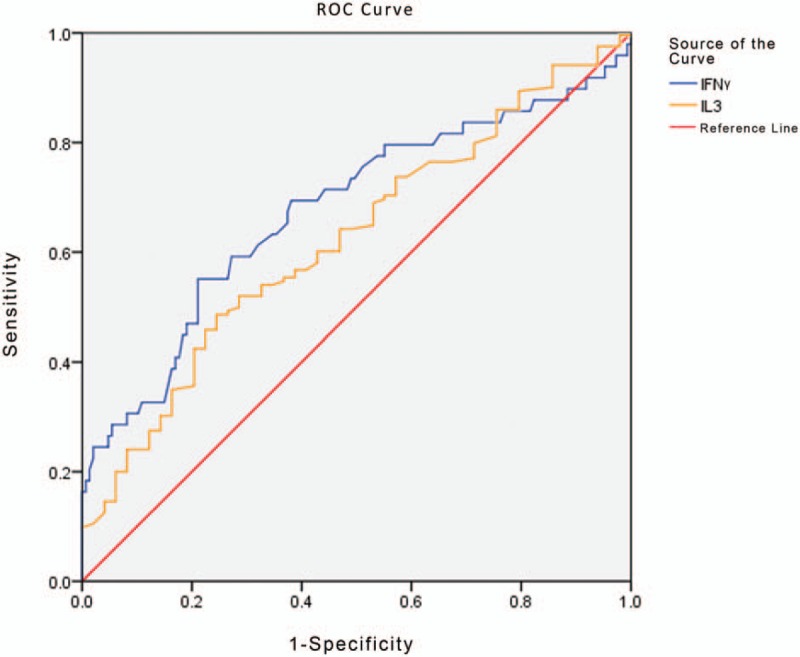
ROC curve analysis showing the differential diagnosis performance of IFN-γ and IL-3 in discriminating GN-BSI from fungal-BSI. The AUCs of IFN-γ and IL-3 were 0.677 and 0.621, respectively. AUC = area under curve, fungal-BSI = Fungal bloodstream infection, GN-BSI = gram-negative bloodstream infection, IFN-γ = interferon-γ, IL-3 = interleukin-3 ROC = receiver-operating characteristic.

IL-3 and IFN-γ were examined for the differential diagnosis of GN-BSI and fungal-BSI (Fig. 3). The analysis showed that the AUC was 0.677 when the cutoff value of IFN-γ was 22.47 pg/mL, and the AUC for IL-3 at a cutoff value of 1.35 pg/mL was 0.621. These 2 markers demonstrated similar differential diagnostic efficacy (Table 6).

**Table 6:**
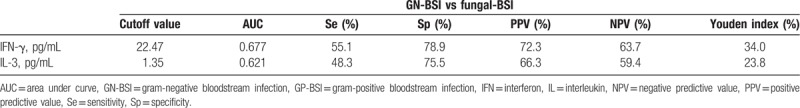
Values of biomarkers in discriminating GN-BSI from fungal-BSI.

**Figure 4:**
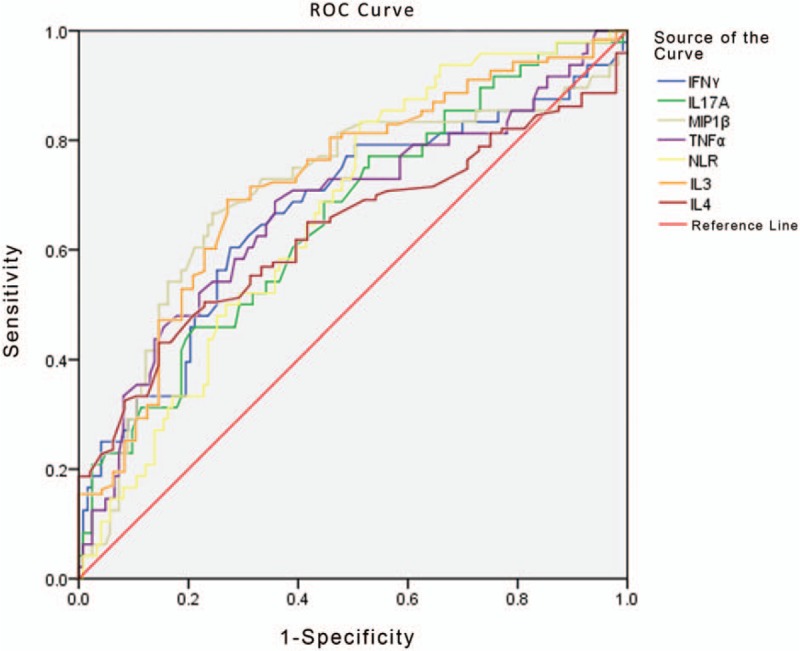
ROC curve analysis showing the differential diagnosis performance of IL-3, MIP-1β, TNF-α, IFN-γ, NLR, IL-17A, and IL-4 in discriminating fungal-BSI from GP-BSI. The AUCs of IL-3, MIP-1β, TNF-α, IFN-γ, NLR, IL-17A, and IL-4 were 0.722, 0.703, 0.673, 0.670, 0.667, 0.658 and 0.632 respectively. AUC = area under curve, fungal-BSI = fungal bloodstream infection, GP-BSI = Gram-positive bloodstream infection, IFN-γ = interferon-γ, IL-17A = interleukin-17A, IL-3 = interleukin-3, IL-4 = interleukin-4, MIP-1β = macrophage inflammatory protein 1β, NLR = neutrophil lymphocyte ratio, ROC = receiver-operating characteristic, TNF-α = tumor necrosis factor α.

Seven markers, IL-3, IL-4, MIP-1β, TNF-α, IFN-γ, NLR, and IL-17A, were selected for examination of the differential diagnosis between Fungal-BSI and GP-BSI (Fig. 4). IL-3 showed better diagnostic efficacy than other indicators. When the cutoff value was 1.17 pg/mL, IL-3 had an AUC of 0.722, followed by MIP-1β with an AUC of 0.703 when the best cutoff value was 61.44 pg/mL (Table 7).

**Table 7:**
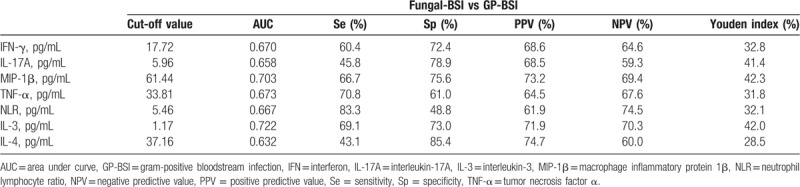
Values of biomarkers in discriminating fungal-BSI from GP-BSI.

The level of IL-3 in the GP-BSI group was significantly higher than those in the other 3 groups (Tables 3 and 4, Fig. 1). By ROC analysis, IL-3 also showed some predictive efficacy in differentiating GP-BSI from GN-BSI and in differentiating GP-BSI from fungal-BSI (Tables 5 and 7, Figs. 2 and 4). So, IL-3 may be a valuable marker for the prediction of GP-BSI.

The level of IFN-γ in the fungal-BSI group was significantly higher than those in the other 3 groups (Tables 3 and 4, Fig. 1). By ROC analysis, IFN-γ also showed some predictive efficacy in differentiating fungal-BSI from GN-BSI and in differentiating fungal-BSI from GP-BSI (Tables 6 and 7, Figs. 3 and 4). Hence, IFN-γ may be a valuable marker for the prediction of Fungal-BSI.

NLR, MIP-1β, IL-3, and IFN-γ were selected for the prediction and validation experiments on clinical patient samples. The results showed that when NLR≧8.25, GN-BSI was much more likely to occur than GP-BSI (Table 8). When serum MIP-1β level was ≧61.44 pg/mL, GN-BSI or fungal-BSI was much more likely to occur than GP-BSI (Tables 8 and 9). When IFN-γ level was ≧22.47 pg/mL, the predictive ratio of fungal-BSI was 80.0% (Tables 10 and 11), and when IL-3 level was ≧1.35pg/mL, the predictive ratio of GP-BSI was 73.2% (Table 11).

**Table 8:**
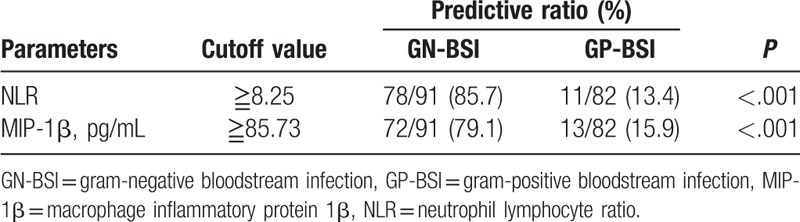
Predictive ratio of NLR and MIP-1β for GN-BSI and GP-BSI.

**Table 9:**
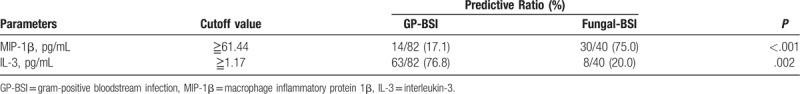
Predictive ratio of IL-3 and MIP-1β for GP-BSI and fungal-BSI.

**Table 10:**
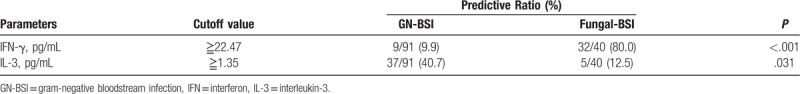
Predictive ratio of IFN-γ and IL-3 for GN-BSI and fungal-BSI.

**Table 11:**
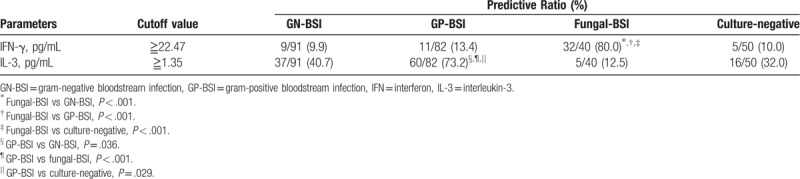
Predictive ratio of IFN-γ for Fungal-BSI and predictive ratio of IL-3 for GP-BSI.

## 4 Discussion

This study found that NLR and MIP-1β may be valuable in differentiating GN-BSI from GP-BSI in hospitalized patients. IFN-γ and IL-3 may be helpful in differential diagnosis GN-BSI and fungal-BSI. IL-3 and MIP-1β exhibited some diagnostic efficacy in distinguishing fungal-BSI and GP-BSI. Additionally, IL-3 with high serum level may be a marker for GP-BSI and IFN-γ with high serum level may be a valuable marker for the prediction of fungal-BSI.

In recent years, because of the abuse of antibiotics, the unnecessary use of intravascular catheters and the increasing development of invasive diagnostic techniques, the incidence of BSI has remained high. BSIs develop quickly and have a mortality rate as high as 30%. Therefore, early, rapid, accurate, and reliable differential diagnosis of different types of BSIs plays a crucial role in the treatment outcome and can improve the survival rate and prognosis of patients.^[15,16]^

Cytokines/chemokines play important roles in infection and anti-infective processes in the body. Stimulation of an immune response against bacteria after they enter the blood is a complex process. Different types of pathogens have various cytokine profiles and contents because of their unique structures and antigenic components.^[17,18]^ Unlike IL-1, IL-6, IL-8, and IL-10, which have been widely studied, IL-3, IL-4, IL-12p70, IL-17A, MIP-1β, and IFN-γ are rarely been reported in differentiating different types of BSIs. Moreover, our previous animal experiments have shown that the serum levels of these cytokines/chemokines were different in mice with different types of BSIs. As a marker of inflammation, the role of P-selectin in determining sepsis patients in the emergency department has been reported sporadically.^[19]^ Hence, IL-3, IL-4, IL-12p70, IL-17A, MIP-1β, IFN-γ, and P-selectin, together with 2 classic inflammatory markers, TNF-α and NLR, were selected for study.

In this study, the levels of 9 selected markers among different groups were compared firstly, and the results showed that the IL-12p70 and P-selectin levels did not significantly differ among the GN-BSI, GP-BSI, and fungal-BSI groups (*P* > .05). Therefore, we focused on evaluation of the diagnostic efficacy of other 7 factors, IL-3, IL-4, MIP-1β, TNF-α, IFN-γ, NLR, and IL-17A, for different BSI types.

According to research reports, compared with patients with GP-BSI, patients with GN-BSI have more serious inflammatory reaction, which is manifested as more serious clinical conditions.^[20]^ In this study, when distinguishing GN-BSI and GP-BSI, IL-3, NLR, MIP-1β, TNF-α, and IL-17A showed some differential diagnostic efficacy and the NLR had the best predictive power, followed by MIP-1β. NLR has been reported as an easily measurable parameter that evaluates the severity of systemic inflammation and sepsis,^[21]^ and it has also been advised to be a valuable predictor of bacteremia.^[22,23]^ However, there is a lack of information about the potential use of NLR to discriminate GN-BSI from GP-BSI and fungi-BSI in hospitalized patients. This study showed that the AUC for NLR (cut-off = 8.25) was greater than those for MIP-1β, TNF-α, IL-17A, and IL-3 when distinguishing GN-BSI and GP-BSI; however, the AUC for NLR (cut-off = 5.46) was less than IL-3, MIP-1β, TNF-α, and IFN-γ, respectively, when distinguishing fungi-BSI and GP-BSI. As a chemokine, MIP-1β can chemotaxis monocytes to the inflammatory site, thus exerting its anti-inflammatory effect. Our previous experiments on BALB/c mice found that MIP-1β level increased in the early stage after bacteria entering the blood, and the higher the level of elevation, the more likely it is to indicate the occurrence of GN-BSI. This study confirmed that the level of MIP-1β was significantly higher in GN-BSI group than in GP-BSI group (*P* < .001).^[24]^ The results also showed that the level of MIP-1β in the Fungal-BSI group was significantly higher than that in the GP-BSI group (*P* < .001). MIP-1β also showed some diagnostic efficacy in distinguishing between fungal-BSI and GP-BSI with an AUC of 0.703 when the best cut-off value was 61.44 pg/mL.

In the study, the levels of IL-4, IL-12p70, IL-17A, MIP-1β, NLR, P-selectin, and TNF-α did not significantly differ between GN-BSI and Fungal-BSI group. Therefore, we focused on the differential diagnostic value of IL-3 and IFN-γ in distinguishing between GN-BSI and fungal-BSI.

Interestingly, the level of IL-3 in the GP-BSI group was significantly higher than those in the GN-BSI and fungal-BSI group (*P* = .022 and *P* < .001). IL-3 showed some predictive efficacy in differentiating GP-BSI from GN-BSI and in differentiating GP-BSI from fungal-BSI. In the prediction experiments, when IL-3 level was ≧1.35pg/mL, the predictive ratio of GP-BSI was 73.2%. So, IL-3 may be a valuable marker for the prediction of GP-BSI.

As an important upstream cytokine in inflammation, IL-3, also named multicolony-stimulating factor (MSF), plays a crucial role during sepsis; however, its exact role is unclear. IL-3 is derived from the innate response activating factor B cells, mast cells, T cells, and eosinophils, and contributes to leukocyte and monocyte production, proliferation, and survival.^[25,26]^ When pathogen invasion occurs, high concentrations of IL-3 could lead to sharp increase in the number of mononuclear cells in circulation, and would result in the excessive release of IL-1β, TNF-α, and IL-6, which have been confirmed as key pro-inflammatory cytokines in sepsis, triggering inflammatory cytokines cascade response and exacerbating the severity of sepsis.^[27–30]^ The role IL-3 plays in the progress of BSI is not entirely clear. Sashida^[31]^ reported that Staphylococcal phage lysates and peptidoglycan subunits of *Staphylococcus epidermidis* could significantly stimulate the replication of spleen monocytes. The stimulated spleen monocytes produce IL-3 and colony-stimulating factor. This may partly explain why IL-3 levels increase significantly in GP-BSI. More pathological mechanism and clinical evidence need to be further obtained.

The level of IFN-γ in the fungal-BSI group was significantly higher than those in the GN-BSI and GP-BSI group (*P* < .001 and *P* = .001). IFN-γ is a pluripotent cytokine that has been shown to play an important role in the function of almost all immune cells and the innate and adaptive immune responses. Its pathophysiological mechanism, antitumor activity, antiinfection activity, and therapeutic potential have been extensively studied. In the early stage of host infection, natural killer (NK) cells and NKT cells secrete interferon gamma, promoting the recruitment and activation of immune cells. IFN-γ also activates NK cells and NKT cells, enhancing their cytotoxicity and cell-mediated immune response.^[32,33]^ Fungal-BSI is an increasingly common and serious problem. The most common pathogens are candida and aspergillus. Studies have shown that when fungal infections occur, high levels of IFN-γ increase the anti-fungal activity of macrophages and neutrophils, which is beneficial to the body in clearing pathogens. IFN-γ has been used as an adjunctive treatment for immunocompromised patients (such as leukemia patients, HIV patients, and transplant patients) with invasive fungal infections.^[34,35]^ This may be the reason for fungal-BSI patients have significantly higher levels than GN-BSI and GP-BSI patients. In our study, IFN-γ showed some predictive efficacy in differentiating fungal-BSI from GN-BSI and in differentiating fungal-BSI from GP-BSI. Hence, IFN-γ may be a valuable marker for the prediction of fungal-BSI.

Our results also suggested that TNF-α and IL-17A have moderate diagnostic ability when distinguishing between GN-BSI and GP-BSI, and TNF-α, IL-17A, and IL-4 have moderate diagnostic ability when distinguishing between GP-BSI and fungal-BSI. In addition, the levels of P-selectin and IL-12p70 were not significantly different among the GN-BSI, GP-BSI and fungal-BSI patients (*P* > .05), despite that previous studies have occasionally reported that P-selectin and IL-12p70 play an important role in the regulating immune activation and participating in inflammatory response when infection occurs.^[36,37]^ Similar investigations of these factors need to be performed in the future.

This study has several limitations. First, up to now, it was difficult to distinguish between contaminants or infections unknown and real infections based on blood culture results only. In our study, 24 culture results indicating CoNS together with 54 other contaminants were excluded, which may generate some selection bias. Second, a low number (n = 49) of hospitalized patients with fungal infection were enrolled, reflecting the low prevalence of fungal BSI. Another limitation was that the predictive efficacy of each single indicator was assessed, but the value of combinations of ≥2 factors was not evaluated, and this should be taken into account in future studies.

## Author contributions

**Conceptualization:** Chengbin Wang.

**Data curation:** Xinjun Li, Xiaozhou Yuan.

**Formal analysis:** Xinjun Li, Xiaozhou Yuan.

**Funding acquisition:** Chengbin Wang.

**Investigation:** Xinjun Li, Xiaozhou Yuan, Chengbin Wang.

**Methodology:** Xinjun Li, Xiaozhou Yuan.

**Project administration:** Chengbin Wang.

**Resources:** Chengbin Wang.

**Software:** Xinjun Li, Xiaozhou Yuan.

**Supervision:** Chengbin Wang.

**Validation:** Xiaozhou Yuan, Chengbin Wang.

**Visualization:** Xinjun Li.

**Writing – original draft:** Xinjun Li, Xiaozhou Yuan.

**Writing – review & editing:** Chengbin Wang.
